# Perceptions and attitudes of medical students towards student evaluation of teaching: A cross-sectional study

**DOI:** 10.1080/10872981.2023.2220175

**Published:** 2023-06-04

**Authors:** Abdulkarim S. Almakadma, Nader A. Fawzy, Omar J. Baqal, Sudha Kamada

**Affiliations:** a College of Medicine, Alfaisal University, Riyadh, Saudi Arabia; b Department of Internal Medicine, Mayo Clinic Arizona, Phoenix, AZ, USA; c Department of Medical Oncology, All India Institute of Medical Sciences, Bhubaneswar, India

**Keywords:** student evaluation of teaching, faculty evaluation, medical education, medical students, course evaluation

## Abstract

**Background:**

Faculty evaluation surveys in the frame of student evaluation of teaching (SETs) are a widely utilized tool to assess faculty teaching. Although SETs are used regularly to evaluate teaching effectiveness, their sole use for making administrative decisions and as an indicator of teaching quality has been controversial.

**Methods:**

A survey containing 22 items assessing demographics, perception, and factors for evaluating faculty was distributed to medical students at our institute. Statistical analyses were conducted using Microsoft Excel and R Software utilizing regression analysis and ANOVA test.

**Results:**

The survey received 374 responses consisting of 191 (51.1%) male students and 183 (48.9%) female students. In all, 178 (47.5%) students considered the optimal time for providing faculty evaluation to be after the release of the exam results, compared to 127 (33.9%) students, who chose the after the exam but before the release of exam results option. When asked what happens whenever the tutor is aware about the SETs data, 273 (72.9%) and 254 (67.9%) students believed that it would influence the difficulty of the exam and grading/curving of the exam results, respectively. Better teaching skills (93%, 348), being responsive and open to student feedback and suggestions (84.7%, 317), being committed to class time and schedule (80.1%, 300), and an easier exam (68.6%, 257) were considered important factors to acquire a positive evaluation by a considerable proportion of students. Fewer lectures (*P* < 0.05), decreased number of slides per lecture (*P* < 0.01), easier exam (*P* < 0.05), and giving clues to students about the exam (*P* < 0.05) were found to be very important to obtain a positive tutor evaluation by students.

**Conclusions:**

Institutions ought to continue exploring areas of improvement in the faculty evaluation process while raising awareness among students about the importance and administrative implications of their feedback.

## Introduction

Faculty evaluation surveys in the frame of student evaluation of teaching (SETs) are a widely utilized tool to assess faculty teaching. It forms the basis for administrative decisions ranging from changes to the curriculum to the promotion of personnel and even the allocation of funds or grants [1,2]. The information collected is invaluable as students can assess various aspects of the educational environment and provide the administration with valuable and valid insight for improving the learning process. Although SETs are regularly used as a reference for teaching effectiveness, their sole use particularly for making administrative decisions and as an indicator of teaching quality has been controversial [3,4]. Withholding the grades until students complete the SETs, a practice in many institutions globally to maintain high response rates, has put the validity of the responses in question. Plus, the survey results are very malleable; for example, the provision of chocolate cookies during sessions was found to have a significant impact on the evaluation received [5,6]. In a multi-instructor course, students were required to submit an anonymous rating of all lecturers using a Likert scale and had the option to not evaluate a lecturer [7]. However, the survey deliberately contained one fictitious lecturer, and results clearly showed that the students filled out the survey mindlessly even when provided with a photograph of an individual that never taught them. In a review of the literature on SETs, it was concluded that the averages of students’ responses to questions concerning effectiveness do not measure teaching effectiveness [8].

On the other hand, implementing SETs has increased university grade point averages, although paradoxically students appear to spend less time on their studies. This was more attributed to faculty leniency with less workload and higher grades, to conform to the student’s needs in order to improve their teaching evaluations, rather than their teaching effectiveness [9,10]. As a result, the responses will mostly be positive, and this phenomenon is especially evident in institutions that heavily base personnel decisions on student evaluations. Ironically, students who rated low SET scores to a certain professor in the first semester were more likely to score better in the second semester [11]. A possible explanation for this phenomenon is that skilled professors achieve a certain level of academic persistence which the students like less but learn more from. Many variables unrelated to teaching effectiveness play a role in SET scores, including the instructor’s race, age, gender, physical attractiveness; student’s grade expectation, enjoyment of the class, and even the weather of the day the survey is completed [12–14]. Even gender bias and grade expectation were found to be more sensitive indicators of SET scores rather than teaching effectiveness. This further resonates with the idea that SETs are not reliable for measuring teaching effectiveness [15].

Nonetheless, numerous methods attempting to minimize the drawbacks of conventional end-of-course surveys have been investigated. For example, Small Group Instructional Diagnosis (SGIDs), is an informal mid-semester evaluation of courses where a facilitator asks questions to students and then divides them into small groups where they can come up with answers together [16]. Afterward, the class can vote on the most critical factors, which will be referred to the course instructor privately. Another study employed sampling for SETs and reported preserved reliability with an increase in the validity of the mean scores and reduced demands on students [17].

Through this study, we investigate the perceptions and attitudes of medical students towards SETs, while exploring the factors they value most as part of this process. Notably, to the best of our knowledge, ours is the first such study to emerge from the Middle East and one of the few globally investigating student attitudes towards SET in the context of medical education.

## Materials and methods

### Survey development

Our survey encompassed 22 items: 6 items assessing demographics, 4 items evaluating perception, and 12 items concerning factors considered when evaluating faculty (supplementary material 1). The key topics covered in the questionnaire were: the importance of faculty evaluation, the influence of evaluation being known to the faculty on exam difficulty and grading, the optimal time of administration of faculty evaluation forms, and factors taken into consideration when evaluating faculty. In the perception assessment section, the survey included one 3-point Likert scale question and three closed-ended questions. In the factors assessment section, 12 factors were appraised using the 3-point Likert scale in the format of a table. The Likert scale options consisted of not at all important [1], neutral [2], and important [3]. Finally, the survey concluded with one optional open-ended question inviting students to share their comments and recommendations to improve the faculty evaluation process.

To establish instrument validity, the team conducted a literature review to generate the items found in this survey. Moreover, the survey items were evaluated and validated by a panel of experts in the field. A pilot study was conducted on 26 medical students from our university to test the clarity and face validity of the tool and to recognize any technical obstacles. These students were requested in the survey not to fill in the survey again when it is circulated later, to avoid duplication of responses. The pilot study revealed that the average time to complete the survey was around 3–4 minutes. Some of the survey items were modified based on feedback received to improve clarity and prevent ambiguity. In addition, there were a few technical issues encountered in the factors table, all of which were fixed before the distribution of the survey which was created on and shared on Google© Forms.

### Study population

The study population consisted of medical students from all academic years studying at the College of Medicine at our university. University faculty and staff, students who have already graduated, and non-medical students were excluded from the study population.

### Survey distribution

A message bearing the questionnaire link was circulated via the university’s institutional email system to the target population. The survey contained an opening paragraph that describes the study’s aim and confirmed participant anonymity as well as the liberty to withdraw or decline a response entirely.

### Statistical analysis

The results of our study were analyzed by using Microsoft Excel® and R Software© using regression analysis and ANOVA test. Significance was adopted at *p* < 0.05 for the interpretation of the results of tests of significance.

### Ethics approval

In compliance with the provisions of the Law of Ethics of Research on Living Creatures and Regulations, and under the guidelines of the National Committee of Bioethics, ethical approval has been acquired from the university’s Institutional Review Board, Reference IRB-20017. Informed consent was obtained from all students.

## Results

The survey received 374 responses from our medical students’ body which encompasses international students from 39 nationalities. Survey respondents’ characteristics are described in Table 1. Our study consisted of 191 (51.1%) male students and 183 (48.9%) female students. Among all academic years, year 4 students had the highest response rate with 88 (23.5%) students. The cumulative GPA of 310 (82.8%) students who participated in the study was 3.1–4.0 out of 4.0.

**Table 1.** Distribution of study population.

Variable	Study population (*n*=374)	Total (%)
**Gender**		
Female	183	48.9
Male	191	51.1
**Current academic year**		
1	70	18.7
2	67	17.9
3	64	17.1
4	88	23.5
5	37	9.8
6	48	12.8
**Cumulative GPA**		
1.5–2.0	6	1.6
2.1–2.5	12	3.2
2.6–3.0	46	12.2
3.1–3.5	155	41.4
3.6–4.0	155	41.4

### Perception towards providing SETs

1. When asked about the importance of providing faculty evaluation, 242 (64.6%) students answered ‘important’.

(a) 44 (62.8%), 43 (64.1%), 40 (62.4%), 62 (70.4%), 27 (72.9%), and 26 (54.1) students in academic years 1, 2, 3, 4, 5, and 6, respectively.

2. It was the most popular opinion, by 178 (47.5%) students, that the optimal time for providing faculty evaluation is after the release of the exam results, compared to 127 (33.9%) students, who chose after the exam but before the release of exam results, and 69 (18.4%) students, who chose entirely before the exam.
3. 273 (72.9%) and 254 (67.9%) students believed that the knowledge of the tutor about faculty evaluation data would influence the difficulty of the exam as well as the grading/curving of the exam results, respectively

## Factors considered by students when filling out SETs

Various factors were included in the survey that are considered by students when filing out faculty evaluation surveys. The findings are summarized in Table 2, with significant results of the Chi-square test in Table 3. Of these, the significant findings were:

1. Better teaching skills (93%, *n* = 348), being responsive and open to student feedback and suggestions (84.8%, *n* = 317), being committed to class time and schedule (80.2%, *n* = 300), and easier exams (68.6%, *n* = 257) were regarded as important factors to obtain a positive evaluation by a considerable proportion of students.
2. Fewer lectures (*p* < 0.05), decreased number of slides per lecture (*p* < 0.01), easier exam (*p* < 0.05), and giving clues to students about the exam (*p* < 0.05) were found to be very important to obtain a positive evaluation by students.
  - Female (*p* < 0.01) and academic year 1 (*p* < 0.05) students were more likely to consider both decreased number of slides per lecture and easier exam as very important for a positive evaluation of faculty.
  - When asked about the importance of giving clues to students about exams, academic year 1 students were more likely to consider it very important for a positive evaluation of faculty (*p* < 0.05).

**Table 2.** Factors considered by students for a positive evaluation in SETs.

Factors	Not at all important	Neutral	Important	p-value
Higher English proficiency	34 (9.1)	81 (21.7)	259 (69.3)	.638
Easier exams	49 (13.1)	68 (18.2)	257 (68.7)	.049
Fewer lectures	69 (18.4)	103 (27.5)	202 (54.0)	.0246
Decreased number of slides per lecture	48 (12.8)	86 (23.0)	240 (64.2)	.000244
Giving clues to students about exam	73 (19.5)	82 (21.9)	219 (58.6)	.0244
Being committed to class times and schedule	25 (6.7)	49 (13.1)	300 (8.2)	.0771
Being lenient about attendance	232 (62.0)	84 (22.5)	58 (15.5)	.00828
Being lenient about class discipline	85 (22.7)	109 (29.1)	180 (48.1)	.749
Better teaching skills	7 (1.9)	19 (5.1)	348 (93.0)	.186
Better appearance (physical, clothing)	191 (51.1)	80 (21.4)	103 (27.5)	.385
Being responsive and open to student feedback and suggestions	17 (4.5)	40 (10.7)	317 (84.8)	.559
Tutor relationship with the student (extracurricular, research, same ethnicity, personal relationship, etc)	71 (19.0)	81 (21.7)	222 (59.4)	.697

Note: **Numbers reported as n (%), *n* = 374**.

**Table 3.** Significant results of Chi-square test for factors considered by students for a positive evaluation in SETs.

Variable		Not at all important	Neutral	Important
**Being lenient about attendance**				
Current Academic Year **(*p* = 0.0205)**	1 (*n* = 70)	42 (60.0)	16 (22.9)	12 (17.1)
	2 (*n* = 67)	46 (71.9)	8 (11.9)	13 (19.4)
	3 (*n* = 64)	46 (71.9)	13 (20.3)	5 (7.8)
	4 (*n* = 88)	56 (63.6)	19 (21.6)	13 (14.8)
	5 (*n* = 37)	15 (4.5)	13 (35.1)	9 (24.3)
	6 (*n* = 48)	27 (56.3)	15 (31.3)	6 (12.5)
**Being committed to class times and schedule**				
Gender **(*p* = 0.0191)**	Female (*n* = 183)	8 (4.4)	21 (11.5)	154 (84.2)
	Male (*n* = 191)	17 (8.9)	28 (14.7)	146 (76.4)
**Decreased number of slides per lecture**				
Gender **(*p* = 0.00932)**	Female (*n* = 183)	18 (9.8)	39 (21.3)	126 (68.9)
	Male (*n* = 191)	30 (15.7)	47 (24.6)	114 (59.7)
Current Academic Year **(*p* = 0.0365)**	1 (*n* = 70)	10 (14.3)	9 (12.9)	51 (72.9)
	2 (*n* = 67)	9 (13.4)	16 (23.9)	42 (62.7)
	3 (*n* = 64)	7 (1.9)	20 (31.3)	37 (57.8)
	4 (*n* = 88)	9 (1.2)	18 (20.5)	61 (69.3)
	5 (*n* = 37)	4 (1.8)	6 (16.2)	27 (73.0)
	6 (*n* = 48)	9 (18.8)	17 (35.4)	22 (45.8)
**Easier exam**				
Gender **(*p* = 0.00354)**	Female (*n* = 183)	18 (9.8)	28 (15.3)	137 (74.9)
	Male (*n* = 191)	31 (16.2)	40 (20.9)	120 (62.8)
Current Academic Year **(*p* = 0.00114)**	1 (*n* = 70)	5 (7.1)	7 (1.0)	58 (82.9)
	2 (*n* = 67)	17 (25.4)	11 (16.4)	39 (58.2)
	3 (*n* = 64)	7 (1.9)	18 (28.1)	39 (6.9)
	4 (*n* = 88)	6 (6.8)	16 (18.2)	66 (75)
	5 (*n* = 37)	7 (18.9)	6 (16.2)	24 (64.9)
	6 (*n* = 48)	7 (14.6)	10 (20.9)	31 (64.6)
**Giving clues to students about exam**				
Current Academic Year **(*p* = 0.0295)**	1 (*n* = 70)	8 (11.4)	15 (21.4)	47 (67.1)
	2 (*n* = 67)	14 (2.9)	14 (20.9)	39 (58.2)
	3 (*n* = 64)	14 (21.9)	17 (26.6)	33 (51.6)
	4 (*n* = 88)	12 (13.6)	20 (22.7)	56 (63.6)
	5 (*n* = 37)	8 (21.6)	9 (24.3)	20 (54.1)
	6 (*n* = 48)	17 (35.4)	7 (14.6)	24 (5.0)
**Tutor relationship with student**				
Current Academic Year **(*p* = 0.0102)**	1 (*n* = 70)	12 (17.1)	13 (18.6)	45 (64.3)
	2 (*n* = 67)	17 (25.4)	11 (16.4)	39 (58.2)
	3 (*n* = 64)	5 (7.8)	14 (21.9)	45 (7.3)
	4 (*n* = 88)	14 (15.9)	21 (23.9)	53 (6.2)
	5 (*n* = 37)	11 (29.7)	6 (16.2)	20 (54.1)
	6 (*n* = 48)	12 (25.0)	16 (33.3)	20 (41.7)

Note: **Numbers reported as n (%), *n* = 374**.

3. Being lenient about attendance was not at all important for obtaining a positive evaluation by students (*p* < 0.01).
  a. Academic year 2,3, and 4 students were more likely to rate it as being not at all important compared to academic year 1, 5, and 6 students who considered it more important (*p* < 0.05).
4. When asked about the importance of commitment to class times and schedule, female students were more likely to consider t as a very important factor for a positive evaluation of faculty (*p* < 0.05).
5. When asked about the tutor relationship with the student, academic year 1, 2, 3, and 4 students, compared to seniors, were more likely to consider it very important for a positive evaluation of faculty (*p* < 0.01).

## Discussion

SET has been extensively employed in practically all universities globally [18]. Yet, we have limited insight into the evaluation of medical education, considering the especially elaborate construct of medical teaching [4]. Specifically, data is scarce on student perceptions and attitudes toward faculty evaluation. Most institutions conduct faculty evaluation as part of the quality assurance process, considering it a rigid, untouchable part of the process. Often, very little is done to explore room for improvement in the faculty evaluation process, potentially due to the associated cost, time, and lack of expertise for making the needed reform. Even when improvements are planned, implementation often falls through due to a lack of commitment from relevant stakeholders and bureaucratic hurdles.

The principal aim of SET is to aid the faculty to obtain an understanding of the strengths and weaknesses of their teaching and methods of evaluation, and students are aware of the role of SETs in improving the curriculum [19–22]. Still, an argument arises revolving around the competency of students to make judgments, as they may not always be able to evaluate certain areas in SET like course design (objectives, content, methods, assessment), or grading criteria in assessment. Nonetheless, it is generally recognized that students may be the only and most competent source to provide valuable feedback on aspects such as lecture quality, presenting skills of the lecturer, and faculty responsiveness to student concerns [23]. Hence, we must keep in mind that there is an explicit (planning and preparation of the class, knowledge of the subject, the classroom environment, and instruction of teaching) and implicit (importance to core values, the sensibility of the teachers to students, and behavior toward students) curricula when analyzing SET [24].

Our study highlights that there is a general consensus among our students that SET holds importance, particularly amongst more senior students who may attest to the fact that there may have been changes implemented based on SETs. However, implementation of student feedback may take time, and experiencing tangible changes may take longer. In general, students have simple needs and focus on the obvious teaching traits when it comes to evaluating faculty members which include better teaching skills, responsiveness and openness to student feedback and suggestions, and commitment to class time and schedule. A study conducted amongst dental students found similar results [25]. Hence, with a myriad of factors being considered and valued by students, the basics of teaching, punctuality, and flexibility should not be forgotten and could be viewed as important areas for improvement.

It was the most popular choice that the optimal time to provide SETs was after the release of the exam results which could be related to our finding that most students were concerned that the tutors’ awareness of the evaluation submitted by students would influence the difficulty of the exam or change grading/curving of exam results. However, issues of bias arise as students may judge an entire course depending on the perceived difficulty and performance in the exam, giving lenient faculty who include easy questions in the exam better feedback while paying little attention to the quality of teaching. This is further supported by the fact that easier exam difficulty was important for positive evaluation when assessing faculty. Similarly, 40% of faculty members in another study recommended having the SETs at the end of the course compared to mid-course, although students may not necessarily benefit from the changes made in response to their feedback [24]. One of the most common barriers to obtaining feedback from students is the belief that SETs do not result in changes [26,27]. Hence, a more transparent environment needs to be pursued with the students, clearly demonstrating how the SET results can lead to planned changes to improve the course.

In general, students tend to provide lenient professors – those who provide them with easy and brief lectures, and simple questions in the exam – with better feedback on SET. It was observed that professors engaged in lax grading practices for self-benefit, as to acquire positive remarks in students’ evaluation, which in turn would help them with promotion and tenure [28,29]. If students were to be given relatively difficult lectures and questions in the exam, a greater number would underperform which may reflect negatively on professors and their teaching abilities. Moreover, a study conducted in the nursing field found that 59% of tutors lacked confidence to fail students [30]. This poses serious concerns to the integrity of some courses, especially in the healthcare profession where students are expected to be knowledgeable and highly skilled. It was posited that SET feedback is inversely proportional to the performance of students later, which means the higher the SET ratings the poorer the students performed in subsequent courses [31]. This further reiterates the idea that ‘harsh’ and non-lenient professors, who usually receive lower SET ratings for making the course challenging, effectively built a stronger foundation for students to build upon in further courses. Unfortunately, this is a fact to be realized by many administrations too late after considering harsh decisions against the tutor based on the low SET ratings. In specific, first-year students were significantly more likely to consider the length of lectures, exam difficulty, and giving clues about exams as important factors when assessing professors. First-year students, who are more likely to possess greater levels of stress and anxiety, have a desire for the college environment to be the same as the school environment, with lenient teachers and relatively easier exams [32]. However, it is expected as students progress through the years, they will grow in appreciation for the medical curricula and how facing challenging exams and lectures helps them identify areas of personal improvement to build a stronger knowledge base, increasing the likelihood of future success.

Some medical schools, like ours, impose rules on compulsory lecture attendance for students to be eligible to sit for the final exam, which can make it difficult for students to balance other responsibilities and commitments, such as extracurricular activities (e.g., research, volunteering, etc.) and preparation for medical licensing exams needed for post-graduate medical training. Nonetheless, ‘being lenient about attendance’ was not at all important for students to provide tutors a positive feedback. However, the senior students were more likely to consider it important than the juniors. Usually, senior students at our medical school spend considerable time and effort preparing for medical licensing exams and thus find it helpful for clinical faculty to be lax with attendance. In addition, many senior students are also participating in extracurricular activities and thus a strict attendance policy adds further time constraints.

While the student-tutor relationship was not found to be a significant factor, we noted that more students in academic years 1, 2,3, and 4 considered it very important for faculty to obtain a positive evaluation, as compared to more senior students. Fostering a beneficial connection with faculty can go a long way, from involvement in research projects to letters of recommendation for postgraduate training. Fresh and aspiring junior students, most with minimal experience and involvement, appreciate these relationships more. In contrast, most senior students who are nearing the end of their medical school journey and stepping into the next stage of their careers may not perceive the importance of those relationships as strongly in their position.

We also provided students an opportunity to share their views through an open-ended question on how the faculty evaluation process could be improved. The most common suggestion was not to hold the end-of-semester grades hostage till the feedback evaluation forms are filled out for all courses by the students. The faculty evaluation survey at our institution consists of numerous Likert scale questions followed by a few open-ended questions, all of which are mandatory to answer to be able to submit the form. Some students mentioned that due to their willingness to view their grades, they often answered the same option ‘1’ or ‘5’ on all the Likert scale questions, picking according to their exam performance solely, then filling the open-ended questions with random letters to be able to submit the survey. These disingenuous responses likely significantly influence the overall data obtained. One statement was ‘don’t force us to fill it, we will lie and write anything just to see our grades, make it optional and make us feel that you actually read it’. Making the surveys optional was one of the common suggestions made by students. Rationally, if SETs were optional, it is likely a higher proportion of the responses would be more genuine, thorough, and reflective of the student’s learning experience, even if the total number of responses is lower. Also, one student suggested that attention-check questions be added to the survey to better omit disingenuous responses. Some students were opposed to receiving the surveys at the end of our courses, as that prevents them from directly benefiting from the improvements made to the course based on their feedback.

‘Give us feedback on our feedback so we know whether our evaluation will cause any changes or not’, was another statement we received. Students appear to have a deep-rooted idea that their responses are not being read and there are no changes occurring [33]. ‘Faculty should aim to maximize improvements based on the responses not only to future batches but also to the current batch’, one student mentioned. Making the survey anonymous was also one concern raised by the students. Even though the survey ensures anonymity, students need to log into the institution’s online learning platform with their accounts to fill out the survey. This inhibits students from providing critical feedback.

Students also suggested formal meetings in the form of small group discussions at the end of each course to share feedback. Considering the students’ desire to be heard, having in-person sessions can likely help students give more elaborate feedback and provide important suggestions. Formulating SETs is an art. Analyzing and assessing the interpretive validity of SET scores is also an art [34]. There needs to be better education on the proper way to formulate SETs to obtain as much information as possible that is representative of the real situation. Especially given the high-stakes nature of SETs, it is crucial that they be formulated and analyzed with care.

## Conclusion

Our study offers valuable insight into the application of and students’ experience with SETs in the domain of medical education, especially given the uniqueness of its sociocultural context. Students took into consideration a wide range of factors when filling SETs, from tutor qualities to exam experiences. With SETs suffering from questionable quality and validity of feedback, they should carry less weightage and impact on the administrative decisions, and complement other means of assessing faculty, to provide opportunities for meaningful improvement. Institutions ought to continue exploring different areas for obtaining faculty evaluation while raising awareness among students about the importance and administrative implications of their feedback. Further research is needed to better understand and enhance the faculty evaluation process in the context of medical education.

## Supplementary Material

Supplemental Material

## Declarations

*Ethics approval and consent to participate*

The study protocol was approved by the Institutional Review Board at our College of Medicine (**IRB-20017)**. Informed consent was obtained from all students.
