# Supplementary material for: Perceptions and attitudes of medical students towards student evaluation of teaching: A cross-sectional study
Source: Med Educ Online. 2023 Jun 4;28(1):2220175. doi: 10.1080/10872981.2023.2220175 (PMC10240975; doi:10.1080/10872981.2023.2220175)
Supplement: Supplemental Material [file ZMEO_A_2220175_SM4507.docx]

Supplementary material 1

* Indicates required question

# Gender *

## Mark only one oval.

Female Male

# Current academic year *

## Mark only one oval.

Year 1

Year 2

Year 3

Year 4

Year 5

Year 6 Internship (Medicine)

# Your cumulative GPA (you may round it up to the closest decimal point) *

## Mark only one oval.

3.6-4.0

3.1-3.5

2.6-3

2.1-2.5

1.5-2

# How important do you think it is for you to provide faculty evaluation? *

*Mark only one oval.*

Not at all important

1

2

3

Important

# If your evaluation was known to the tutor, do you think it would inﬂuence the diﬃculty of the exam? *

## Mark only one oval.

Yes No

# If your evaluation was known to the tutor, do you think it would inﬂuence grading and curving of exam results? *

## Mark only one oval.

Yes No

# What is the best timing for you to provide your faculty evaluation? *

## Mark only one oval.

Before exam

After exam and before release of results After release of results

# (ﬂip your phone to landscape mode to view the scales) For each of the following factors, rate how important are they for you to provide a faculty with a positive evaluation in the faculty evaluation surveys? (1= not at all important, 2 = neutral, 3 = important) *

*Mark only one oval per row.*

1 2 3

Better appearance (physical, clothing)

Better teaching skills

Higher English proficiency

Being lenient about attendance

Being lenient about class discipline

Being committed to class times and schedule

Fewer lecture

Decreased number of slides per lecture

Easier exams

Giving clues to students about exam

Being responsive and open to student feedback and suggestions

Tutor relationship with student (extracurricular, research, same ethnicity, personal relationship, etc)

# What recommendations do you have to improve the faculty evaluation process? (Kindly limit to 2 recommendations)
